# Kidney-compartment genomic prioritization across diabetic and population kidney traits: an exploratory Mendelian randomization and colocalization study

**DOI:** 10.3389/fgene.2026.1921914

**Published:** 2026-10-28

**Authors:** Ran Chen, Xiaohui Bi

**Affiliations:** Department of Clinical Laboratory, South China Hospital, Medical School, Shenzhen University, Shenzhen, China

**Keywords:** colocalization, diabetic kidney disease, eQTL, kidney compartments, Mendelian randomization, single-cell transcriptomics

## Abstract

**Introduction:**

Kidney-trait regulatory signals are difficult to prioritize because diabetes-specific and population-wide outcomes, kidney‐compartment eQTL resources, progression phenotypes, disease-state expression data, and locus-level colocalization are usually evaluated separately.

**Methods:**

We assembled an exploratory public-data framework integrating CKDGen, KP4CD, GTEx v10, Susztak/NephQTL, Human Protein Atlas, INTERVAL, Open Targets, and kidney disease single‐cell resources across estimated glomerular filtration rate (eGFR), urinary albumin‐to‐creatinine ratio (UACR), and progression phenotypes. Single-SNP Wald ratios were treated as screening estimates, with primary within-outcome FDR and a study‐wide BH-FDR sensitivity analysis across 597,099 screening rows. Single‐cell, external expression, protein, and tractability resources were used as biological context rather than validation.

**Results:**

GTEx exposure quality control retained 209,031 kidney‐cortex and 2,744,871 whole‐blood significant variant‐gene pairs; outcome‐specific overlap and harmonization yielded 31‐2,232 screening pairs per displayed status outcome. Only UMOD had FDR-level support from diabetes-specific outcomes and general kidney traits. PRKCI and WIPF3 showed robust single-signal approximate Bayes factor colocalization with overall UACR, whereas ITIH4, LINC02166, and A3GALT2 were downgraded because of prior sensitivity or diffuse local patterns.

**Discussion:**

Therefore, the framework prioritizes loci for mechanistic follow‐up while avoiding claims of definitive causal Mendelian randomization, diabetic kidney disease specificity for general-trait signals, or therapeutic readiness.

## 1 Introduction

Diabetic kidney disease (DKD) remains one of the major routes from diabetes to chronic kidney disease (CKD) and kidney failure. Clinical assessment is based on three related but non-identical axes: estimated glomerular filtration rate (eGFR), albuminuria, and subsequent decline in kidney function (Kidney Disease: Improving Global Outcomes CKD Work Group, 2024; American Diabetes Association Professional Practice Committee, 2024). Recent reviews summarize the genetic and epigenetic background of DKD (Roumeliotis et al., 2024; Lyssenko and Vaag, 2023; Sandholm et al., 2023), and large kidney-trait genome-wide association studies (GWAS) have expanded the catalog of kidney-function and albuminuria loci (Cole et al., 2025; Wuttke et al., 2019; Teumer et al., 2019; Teumer et al., 2016). However, these studies have not fully resolved how diabetes-associated regulatory variation maps onto albuminuria, reduced filtration, and progression (Gorski et al., 2021; Robinson-Cohen et al., 2023). Candidate-gene prioritization from transcriptome-wide association and multiomics studies has been useful (Si et al., 2024; Doke et al., 2021; Li et al., 2024), and sequencing studies have added DKD gene discovery (Haukka et al., 2024). Kidney tissue proteomics has also identified matrix metalloproteinase 7 as a biomarker of kidney fibrosis (Hirohama et al., 2023). Many analyses still depend on blood or bulk kidney resources and therefore leave two problems unresolved: which nephron compartment carries the relevant regulatory signal, and whether the same local variant drives both expression and the kidney phenotype.

Four specific gaps motivated this analysis. Diabetic and population-wide kidney outcomes are often examined in separate studies, which makes it difficult to distinguish diabetes-enriched biology from more general kidney biology or ancestry-dependent effects (Cole et al., 2025; Teumer et al., 2016; Robinson-Cohen et al., 2023; Winkler et al., 2022). Progression phenotypes are also frequently handled apart from eGFR and urinary albumin-to-creatinine ratio (UACR), although patients with diabetic kidney disease are managed by both current kidney status and subsequent decline (Gorski et al., 2021; Robinson-Cohen et al., 2023; Klarenbach et al., 2024; Grams et al., 2022). In parallel, human kidney disease atlases (Abedini et al., 2024; Lake et al., 2023) and DKD-focused single-cell studies (Chen et al., 2023; Tsai et al., 2023; Woroniecka et al., 2011; Fan et al., 2019; Liu et al., 2023) have created rich maps of injured kidney cells, but those maps are seldom used as formal components of genetic target prioritization. Finally, Mendelian randomization (MR)-ranked genes are sometimes carried forward without locus-level colocalization, even when expression and disease associations could arise from different linked variants (Skrivankova et al., 2021; Giambartolomei et al., 2014; Wakefield, 2009).

We addressed these gaps by assembling a public-data prioritization framework for kidney dysfunction, albuminuria, and progression in diabetes and the general population. The workflow brought together GTEx kidney-cortex and whole-blood resources (GTEx Consortium, 2020; GTEx Consortium et al., 2017), independent kidney-compartment eQTL data (Qiu et al., 2018), CKDGen and KP4CD outcomes, protein and tractability annotations, disease-state kidney single-cell context, and locus-level colocalization. Outcome scope was retained throughout: diabetes-specific outcomes were labeled separately from overall eGFR, overall UACR, and general CKD progression, and a signal from a general outcome was not reinterpreted as DKD-specific evidence. MR was used to screen genetically proxied gene-expression–kidney-outcome associations. Under the relevance, independence, and exclusion-restriction assumptions, an instrumental-variable estimate may have a causal interpretation; those assumptions were not treated as established here. The objective was deliberately conservative to separate exploratory genetic screening, kidney-compartment reproducibility, disease-cell context, and translational annotation rather than to produce the longest possible list of candidates.

### 1.1 Distinctive features of the present framework

Compared with prior kidney TWAS, multiomics prioritization, and drug-repurposing studies (Si et al., 2024; Doke et al., 2021; Li et al., 2024; Jiang et al., 2025; Chen et al., 2024; Lu and Wang, 2024), this analysis was designed around five features: parallel but explicitly separated diabetes-specific and general kidney-outcome axes, progression phenotypes, independent kidney-compartment reproducibility, disease-state single-cell context, and colocalization-based downgrading of MR-screened candidates with distinct or sensitivity-limited local signals. Therefore, the final panel is a hypothesis-generating follow-up set, not a list of established causal DKD genes.

## 2 Materials and methods

### 2.1 Study design

We constructed a reproducible public-data workflow for diabetes-specific and general kidney traits. The workflow comprised ten analysis components: GTEx whole-blood discovery, GTEx kidney-cortex follow-up, independent kidney-compartment reproducibility, protein annotation, progression outcome integration, healthy kidney single-cell context, disease-state kidney single-cell context, independent bulk disease-expression context, functional and translational annotation, and locus-level colocalization. Disease-state expression, protein, and tractability layers were treated as biological context rather than experimental validation. Source datasets and statistical methods are cited in the relevant subsections below.

### 2.2 Reporting guideline and preregistration

This report was revised in accordance with the Strengthening the Reporting of Observational Studies in Epidemiology using Mendelian randomization (STROBE-MR) guideline and current best-practice guidance for MR investigations (Skrivankova et al., 2021; Burgess et al., 2023). A completed STROBE-MR checklist is supplied as an additional file. The exploratory public-data workflow was not preregistered; the analysis components and thresholds used in the submitted workflow, together with postreview sensitivity additions, are labeled in the manuscript and archived materials.

### 2.3 Outcome summary statistics

Eleven outcome summary-statistics files were standardized to a common schema including chromosome, Genome Reference Consortium Human Build 37 (GRCh37) position, rsID, alleles, effect, standard error, *p*-value, sample size, effect allele frequency, and project variant identifier. They comprised CKDGen diabetic eGFR in European ancestry (n = 14,308) and African ancestry (n = 2,162) (Cole et al., 2025; Winkler et al., 2022); CKDGen overall eGFR (n = 1,201,909) (Wuttke et al., 2019); CKDGen overall UACR in trans-ethnic/all-ancestry analysis (n = 564,257) and European ancestry (n = 547,361) (Teumer et al., 2019); CKDGen diabetic UACR (n = 51,541) (Teumer et al., 2016); CKDGen rapid3 (n = 141,964; 34,874 cases/107,090 controls) and ckdi25 (n = 195,145; 19,901 cases/175,244 controls) progression outcomes (Gorski et al., 2021); and KP4CD diabetes-specific eGFR decline in European ancestry (n = 27,049), mixed ancestry (n = 33,623), and African ancestry (n = 6,574) (Robinson-Cohen et al., 2023). For source files with variant-specific sample sizes, n denotes the source-reported analysis size when provided, or the maximum available sample size in the summary-statistics file. Odds ratios for rapid3 and ckdi25 were converted to log-odds effects during standardization.

### 2.4 eQTL exposure processing

The GTEx v10 kidney cortex (n = 103 genotyped samples) and whole blood (n = 800 genotyped samples) were processed under two exposure definitions: a conventional top-eQTL design and a broader significant-pairs design (GTEx Consortium, 2020; GTEx Consortium et al., 2017). The significant-pairs tables were built from GTEx v10 all-association resources, lifted from GRCh38 to GRCh37, and filtered for valid alleles, unique liftovers, minor allele count ≥ 10, and F statistic > 10. Independent kidney-compartment reproducibility used Susztak/NephQTL tubule (n = 356), glomerulus (n = 303), and kidney-meta (n = 686) eQTL resources; legacy tubule (n = 121) and glomerulus (n = 119) datasets were retained as sensitivity resources (Qiu et al., 2018). These eQTL resources largely represent non-diabetic tissue and predominantly European-ancestry discovery samples; they cannot model diabetes-dependent regulatory effects or provide ancestry-matched exposure estimates for African-ancestry outcomes. Selecting significant eQTLs and then choosing a best instrument can also amplify winner’s-curse bias, especially in the small GTEx kidney-cortex sample, so cross-resource recurrence was used only as supportive screening evidence (Jiang et al., 2023).

### 2.5 Mendelian randomization

Best-instrument MR was used as an exploratory screening layer because kidney eQTL resources often had sparse cis instruments after liftover, allele harmonization, minor-allele-count, instrument-strength, and palindromic-SNP filters. Single-SNP Wald ratios were used for prioritization and direction-aware screening rather than as stand-alone causal effect estimates. Exposure and outcome records were harmonized by chromosome, GRCh37 position, and unordered allele pair; reverse and reverse-complement matches were allowed, and palindromic SNPs were excluded from the default scan. For each gene-outcome-resource combination, one cis instrument was retained using exposure *p*-value, F statistic, and distance to the transcription start site; instrument-strength filtering required F statistic > 10 in the exposure set. This design avoided combining correlated instruments when harmonized LD reference data were not available across all resources, but it cannot provide heterogeneity, horizontal pleiotropy, MR-Egger, weighted-median, or leave-one-out diagnostics. Steiger filtering was not applied because exposure and outcome estimates came from differently scaled summary resources with incomplete variance information; reverse direction and winner’s-curse bias therefore remain unresolved (Hemani et al., 2017; Jiang et al., 2023). Wald-ratio estimates used the aligned outcome effect divided by the exposure effect, with first-order delta approximation for the standard error (Skrivankova et al., 2021; Burgess et al., 2023; Burgess et al., 2013; Smith and Ebrahim, 2003). Primary Benjamini–Hochberg FDR families were defined within each resource-outcome combination; a revision sensitivity analysis additionally applied BH once across all 597,099 screening rows. Representative SNP, F statistic, exposure and outcome beta/SE, harmonization direction, MR beta/SE/CI/P, within-outcome FDR, branch-global FDR, study-wide FDR, and outcome source are reported in Supplementary Data S11 and S12.

### 2.6 Instrumental-variable assumptions and analysis scope

The three core instrumental-variable assumptions are as follows: relevance, meaning that the genetic instrument is associated with the expression exposure; independence, meaning that it is independent of confounders of the expression–outcome relationship; and exclusion restriction, meaning that it affects the outcome only through the expression exposure. Relevance was screened using significant cis-eQTLs and F statistic > 10. Independence and exclusion restriction could not be verified from the available summary data. Cis restriction, harmonization, palindromic-variant exclusion, kidney-compartment recurrence, and colocalization were used as safeguards or locus-context checks, but none establishes these assumptions. A single representative instrument also prevents the assessment of regulatory-variant heterogeneity and multi-instrument pleiotropy. Exposure and outcome coefficients inherited the covariate adjustments used by the original studies; identical covariate sets could not be enforced, and participant overlap between exposure and outcome sources could not be quantified. Records without the required allele, effect, standard-error, or matched-variant fields were excluded, and no individual-level missing-data imputation was performed. No formal *a priori* power calculation was undertaken for this heterogeneous multiresource scan; power varies with outcome and exposure sample size, allele frequency, instrument strength, and precision. Therefore, null or non-retained rows, especially from smaller resources, are not evidence of no effect.

### 2.7 Progression integration

Progression analyses included CKDGen rapid3 and ckdi25 together with KP4CD diabetes-specific eGFR decline in European-, mixed-, and African-ancestry analyses (Gorski et al., 2021; Robinson-Cohen et al., 2023). These outcomes were screened across GTEx kidney-cortex, GTEx whole-blood, Susztak tubule, Susztak glomerulus, kidney-meta, and legacy compartment sensitivity resources. Because the exposure eQTL estimates were not ancestry matched to the African-ancestry outcomes, African-outcome analyses were treated as exploratory transportability checks rather than ancestry-specific replication; LD and allele-frequency differences may affect transferability (Mägi et al., 2017). Progression summaries were consolidated at resource and gene levels.

### 2.8 Protein and functional evidence

Protein evidence was derived from Human Protein Atlas (HPA) kidney immunohistochemistry (IHC) and INTERVAL plasma proteome resources (Karlsson et al., 2021; Uhlen et al., 2015; Sun et al., 2018). The final seven-gene panel was additionally annotated for functional and translational readiness using Open Targets target metadata and tractability annotations, local HPA kidney-IHC evidence, INTERVAL target-assay coverage, and INTERVAL target-protein quantitative trait locus (pQTL) evidence (Sun et al., 2018; Ochoa et al., 2023; Ghoussaini et al., 2021). Genes were classified into translational evidence tiers based on approved-drug, high-quality ligand, druggable-family, antibody or Gene Ontology (GO)-cellular-component, protein-literature, kidney-IHC, target-assay, and target-pQTL evidence.

### 2.9 Single-cell tissue and disease-state context

Healthy-tissue kidney cell-type context was derived from HPA v21 single-cell tissue expression (Karlsson et al., 2021). Disease-state kidney context was extracted from a 2024 multimodal human kidney disease atlas supplementary workbook (Abedini et al., 2024). We used source-reported differential-expression tables for DKD versus control, all disease versus control, and CKD versus control comparisons by cell type, together with source-reported injured proximal-tubule trajectory genes and fibrotic microenvironment gene sets. Source-defined adjusted *p*-value or q-value thresholds were retained; no cross-atlas renormalization, batch correction, or reannotation were performed. A secondary external DKD single-cell supplement was searched as a context sensitivity check for final-panel genes in module, gene-list, ligand-receptor, and pathway-style tables (Tsai et al., 2023). Cell-type labels are reported as provided by the source atlases. Absence from a source list was not treated as a negative experimental result.

### 2.10 Independent expression-context check

To add external disease-expression context, we downloaded processed GEO matrices for GSE30528, GSE30529, and GSE142025 (Woroniecka et al., 2011; Fan et al., 2019). GSE30528 compared microdissected DKD glomeruli (n = 9) with control glomeruli (n = 13). GSE30529 compared DKD tubulointerstitium (n = 10) with control tubulointerstitium (n = 12). For GSE142025, the analyzed processed data comprised kidney biopsy samples from patients with early diabetic nephropathy (DN; n = 6) or advanced DN (n = 21), and control kidney tissue samples (n = 9). For final-panel genes represented in each processed matrix, expression was compared using Welch two-sample t-tests, and BH-FDR was calculated within each dataset and contrast across assayed final-panel genes. Assay coverage, tested but non-significant contrasts, and genes absent from the processed matrices were distinguished explicitly. This layer was used as disease-state expression context and not as genetic, causal, or experimental validation.

### 2.11 Colocalization

Locus-level colocalization was performed using local GTEx v10 all-association parquet shards for the kidney cortex and whole blood (GTEx Consortium, 2020; GTEx Consortium et al., 2017). For each prioritized tissue-locus combination, gene-specific eQTL association data were extracted from the queried locus window, lifted to GRCh37, filtered for valid alleles and unique genomic mappings, allele-aligned to the relevant outcome, and analyzed using Wakefield approximate Bayes factors (ABF) (Giambartolomei et al., 2014; Wakefield, 2009). Posterior probabilities for H0–H4 were estimated using priors p1 = 1e-4, p2 = 1e-4, and p12 = 1e-5. Strong shared-variant-compatible evidence was defined as PP4 ≥ 0.80 under this single-signal model. Dense locus-level association data and matched-LD reference files suitable for harmonized conditional analysis were not available for the Susztak/NephQTL compartment resources in the packaged workflow; therefore, those resources contributed compartment recurrence but not independent colocalization. SuSiE or conditional multisignal colocalization was not performed, although such approaches are preferred when multiple causal variants may be present (Wallace, 2021). *Post*-*hoc* robustness analyses recomputed PP4 from stored locus-level ABF values under alternative shared-variant priors, narrower 500 kb and 200 kb central windows, and SNP-level PP4 concentration with PP3 as a multisignal risk proxy. These checks can flag sensitivity but do not replace LD-aware conditional colocalization.

### 2.12 Statistical thresholds, candidate selection, and reproducibility

Unless otherwise specified, FDR refers to BH adjustment within each resource-outcome, contrast, or displayed result table. Two-sided P-values were used for Wald-ratio screening and Welch tests. The number of rows entering each primary correction is provided in the corresponding source table. A study-wide sensitivity family spanning all 597,099 status and progression screening rows retained 3,137 rows at BH-FDR < 0.05, compared with 5,628 rows under the primary within-outcome families; correlated and partly repeated resource branches mean that this sensitivity analysis is not an independence claim. Colocalization summaries report posterior probabilities from the single-signal Wakefield-ABF model; exact posterior probabilities, prior/window sensitivity, and concentration proxies are retained in Supplementary Data S10.

Candidate selection was stagewise and auditable: exposure QC defined the available eQTL universe; outcome-specific overlap and allele harmonization defined each tested screening family; within-outcome FDR and study-wide sensitivity summarized statistical evidence; kidney-compartment recurrence and diabetes-specific versus general outcome scope were then labeled; biological-context layers were added without being called validation; and colocalization sensitivity determined whether a locus remained primary, was downgraded to secondary, or was excluded. The tested universe, thresholds, retained counts, representative instruments, evidence-scope labels, and downgrade rules are provided in Supplementary Data S11, S12.

### 2.13 Software, reporting resources, and reproducibility

Analyses were executed in a Conda-compatible environment using Python 3.10 and R 4.3. Package versions are recorded in archived environment.yml; dataset identifiers, analysis settings, thresholds, scripts, file checksums, and minimal verification commands are provided in Zenodo archive, revision addendum, and Reproducibility Quickstart. Raw third-party source files remain subject to their original access conditions.

Supplementary data organization. Supplementary Data S1 provides functional and translational context for the final seven-gene panel. Supplementary Data S2 and S3 provide primary and secondary disease-state single-cell context. Supplementary Data S4 and S5 provide resource-level and gene-level progression summaries. Supplementary Data S6 provides gene-level colocalization summaries. Supplementary Data S7 provides the integrated Table 1 source data. Supplementary Data S8 provides MR reporting, data-source access information, and prioritization transparency. Supplementary Data S9 provides independent DKD disease-state expression context. Supplementary Data S10 provides colocalization robustness and specificity analyses. New Supplementary Data S11 provides the diabetes-specific versus general kidney-trait evidence map, representative instrument details, and candidate-selection path. New Supplementary Data S12 provides the study-wide BH-FDR sensitivity universe, resource-outcome counts, and final-panel rows. A reviewer-facing Reproducibility Quickstart lists the direct Zenodo record, archive manifest/checksum, environment setup, and minimal verification commands. An MR reporting summary is supplied as a separate additional file; the same summary is also included in Supplementary Data S8. A completed official STROBE-MR checklist is supplied as an additional reporting-guideline file.

**TABLE 1:** Scope-qualified seven-gene follow-up panel.

Gene	Panel role	Outcome scope	Main support and limitation
*PRKCI*	Primary; albuminuria	General kidney trait	Robust overall-UACR colocalization and compartment context; no FDR-level diabetes-specific support. Tractability annotations are non-DKD context; experimental validation is required.
*WIPF3*	Primary; albuminuria	General kidney trait; nominal diabetic UACR only	Robust overall-UACR colocalization and compartment context; limited translational annotation and no FDR-level diabetes-specific support.
*UMOD*	Primary; progression	Diabetes-specific and general kidney traits	Recurrent compartment support for diabetic eGFR status, KP4CD European/mixed-ancestry decline, and general CKDGen progression; African-ancestry outcome was null, and exposure eQTLs were not ancestry matched.
*ITIH4*	Secondary; eGFR	General kidney trait	Cross-resource overall-eGFR support and disease-state context, but colocalization was prior-sensitive and diffuse; not kidney- or DKD-specific.
*LINC02166*	Secondary; eGFR	General kidney trait	High default overall-eGFR PP4 but prior-sensitive, diffuse, and biologically underannotated.
*A3GALT2*	Secondary; albuminuria	General kidney trait	Default overall-UACR PP4 exceeded 0.80, but strong prior sensitivity, diffuse support, and limited orthogonal annotation downgraded confidence.
*FGF5*	Secondary; progression	General kidney trait	Tubule/glomerular ckdi25 and overall-eGFR support; no FDR-level diabetes-specific progression support and weaker evidence than *UMOD*.

Neutral primary/secondary labels indicate manuscript follow-up priority only, not clinical actionability. Outcome scope distinguishes diabetes-specific evidence from general overall eGFR, overall UACR, or CKD progression evidence. Full layer-by-layer evidence, representative SNPs, F statistics, harmonized effects, P-values, FDR values, outcome sources, and downgrade rules are provided in Supplementary Data S11; the original integrated source data remain in Supplementary Data S7.

### 2.14 Ethics and data-source approval

This study analyzed publicly available or controlled-access summary statistics and released annotation resources only. No new individual-level human participant data, human tissue, or animal experiments were generated; ethics approval and consent for the original studies are described in the source publications and repositories. A full ethics statement is provided in the declarations section.

## 3 Results

### 3.1 Public-data integration expanded kidney-trait coverage

The final analysis covered 11 CKDGen and KP4CD outcomes: six diabetic and overall eGFR and UACR status traits, two general CKDGen progression endpoints, and three KP4CD diabetes-specific eGFR-decline endpoints. Kidney outcomes were connected to molecular QTL resources, exploratory MR screening, biological-context annotations, and colocalization in the study workflow (Figure 1A). After exposure quality control, the GTEx significant-pairs resources contained 209,031 kidney-cortex variant-gene pairs across 2,440 genes and 2,744,871 whole-blood pairs across 13,871 genes. These genome-wide exposure counts are distinct from the outcome-specific denominators in Figure 1B: after outcome overlap, allele harmonization, and instrument filtering, 31–2,232 pairs per displayed status outcome entered the top-eQTL or significant-pairs screening branches. The seven-gene follow-up panel separated into three descriptive axes: *PRKCI*, *WIPF3*, and *A3GALT2* for albuminuria; *ITIH4* and *LINC02166* for eGFR; and *UMOD* with *FGF5* for progression (Figure 1C). Outcome-scope labels in Table 1 and Supplementary Data S11 show that most panel support is derived from general kidney traits rather than DKD-specific outcomes. Best-instrument MR screening evidence across the six status outcomes is summarized in Figure 2.

**FIGURE 1:**
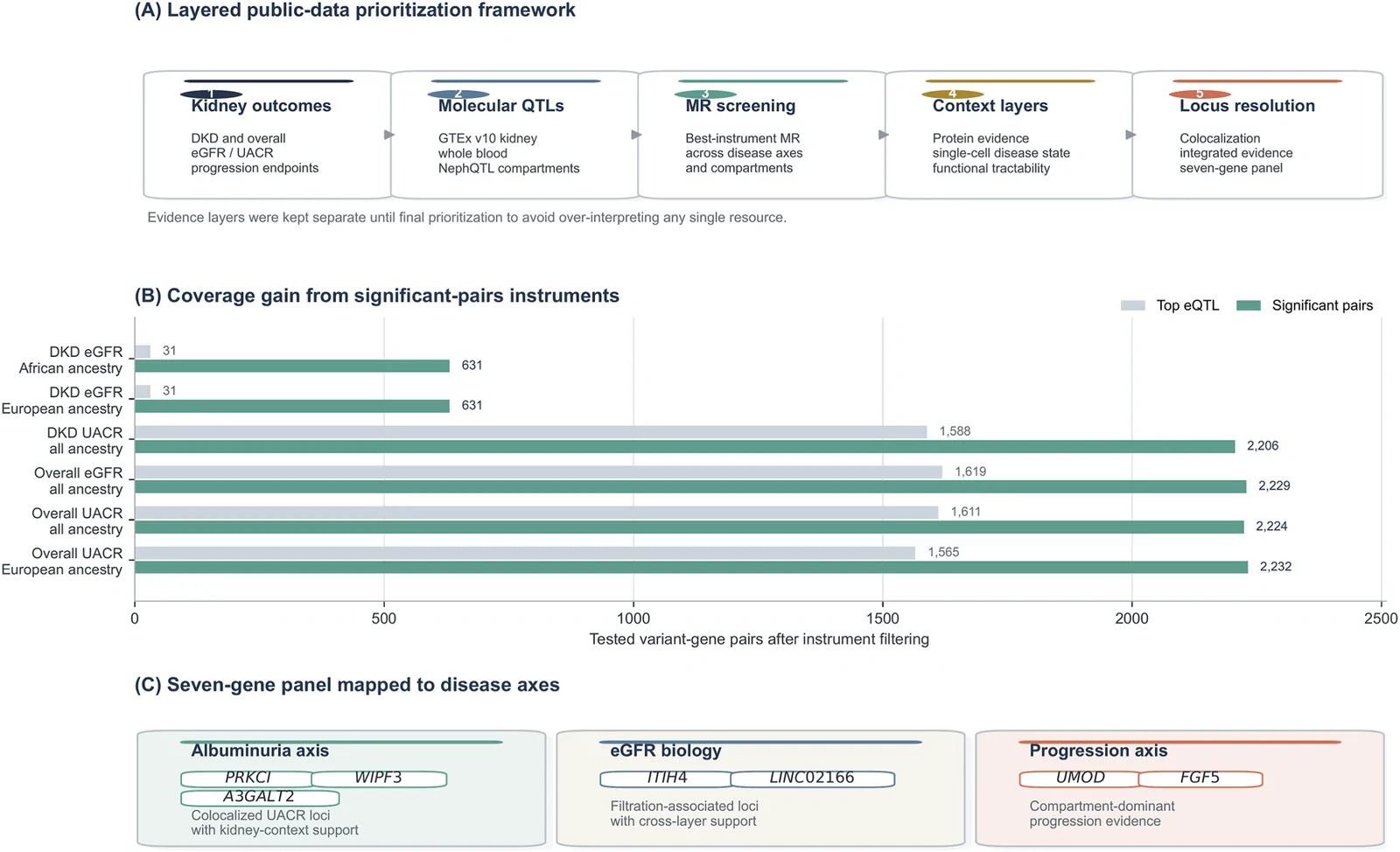
Study design and discovery gain. **(A)** Layered public-data framework linking kidney outcomes, molecular QTL resources, single-SNP MR screening, biological-context layers, and single-signal ABF colocalization before scope-qualified prioritization. **(B)** Counts of outcome-specific variant–gene pairs retained after outcome overlap, instrument filtering, and allele harmonization under top-eQTL and significant-pairs designs (31–2,232 pairs per displayed status outcome). These are not the genome-wide exposure-resource counts of 209,031 kidney-cortex and 2,744,871 whole-blood significant pairs. **(C)** Descriptive disease-axis map of the seven-gene follow-up panel. DM, diabetes mellitus; DKD, diabetic kidney disease; eGFR, estimated glomerular filtration rate; UACR, urinary albumin-to-creatinine ratio; GTEx, Genotype-Tissue Expression; QTL, quantitative trait locus; eQTL, expression QTL; MR, Mendelian randomization.

**FIGURE 2:**
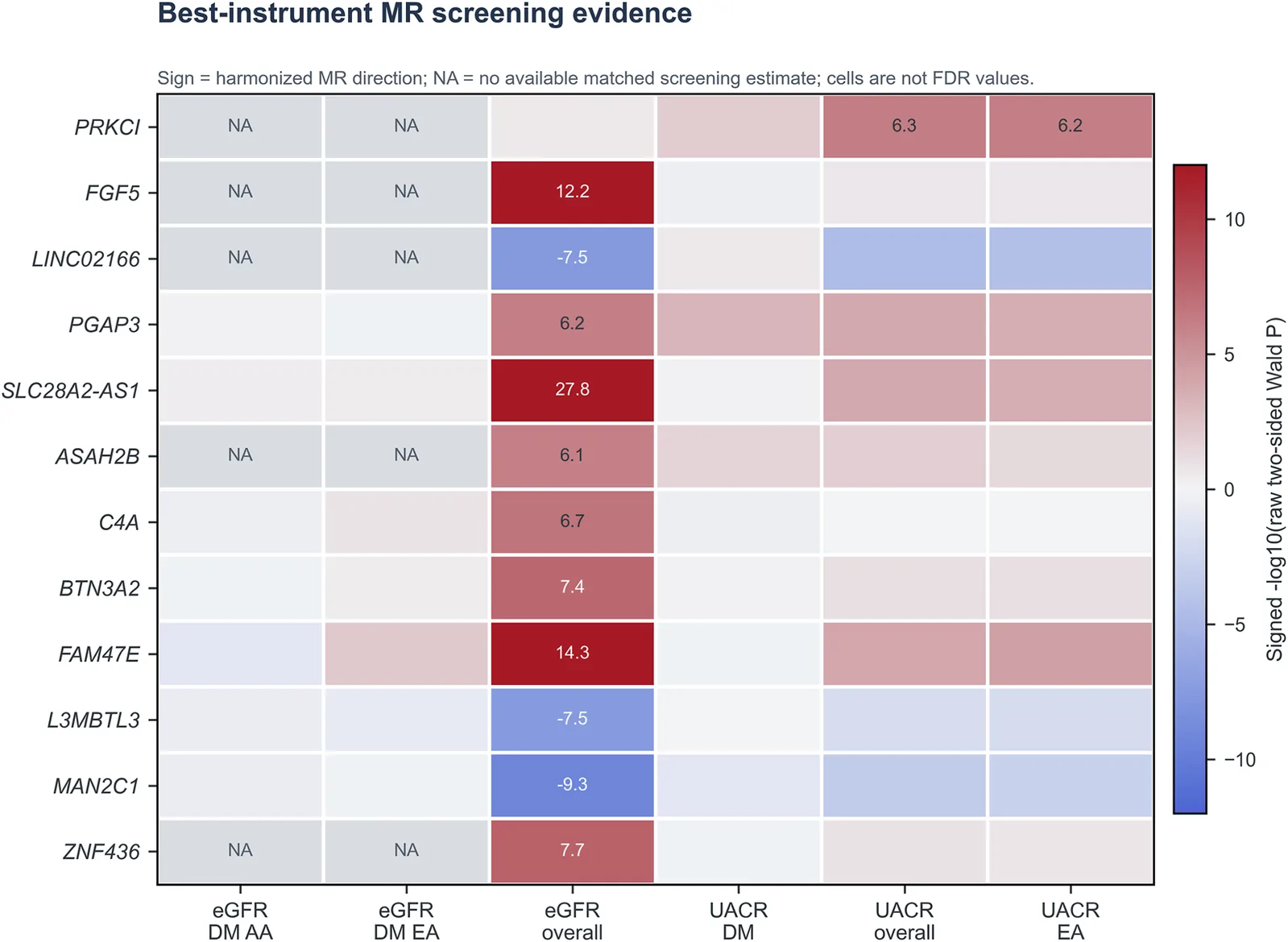
Disease-axis best-instrument MR screening evidence. Heatmap of signed −log10 (raw two-sided Wald-ratio P) screening values across diabetic eGFR in African ancestry; diabetic eGFR in European ancestry; and overall eGFR, diabetic UACR, overall UACR, and overall UACR in European ancestry. The sign is the harmonized MR-estimate direction; red is positive, and blue is negative. Cells are raw P visualizations, not FDR values. Gray cells labeled NA indicate that no matched screening estimate was available after outcome overlap/harmonization; they are not zero effects or non-significant results. MR, Mendelian randomization; DM, diabetes mellitus; AA, African ancestry; EA, European ancestry.

### 3.2 Independent kidney compartments refined the discovery shortlist

Independent kidney-compartment analyses improved cross-resource reproducibility for kidney-context signals, consistent with prior work showing the value of renal compartment-specific molecular QTL analyses (Qiu et al., 2018). The GTEx kidney cortex shared 95, 101, and 132 FDR-level genes with tubule, glomerulus, and kidney-meta resources for overall eGFR, whereas overall UACR showed 6–8 shared FDR-level genes per comparison (Figure 3A). Direct tubule-versus-glomerulus comparison showed that 620 shared FDR-level genes for overall eGFR and 67–80 for overall UACR, depending on outcome definition (Figure 3B). These comparisons reuse kidney-trait outcome summary statistics and therefore represent eQTL-resource reproducibility, not independent external GWAS replication or proof of kidney specificity.

**FIGURE 3:**
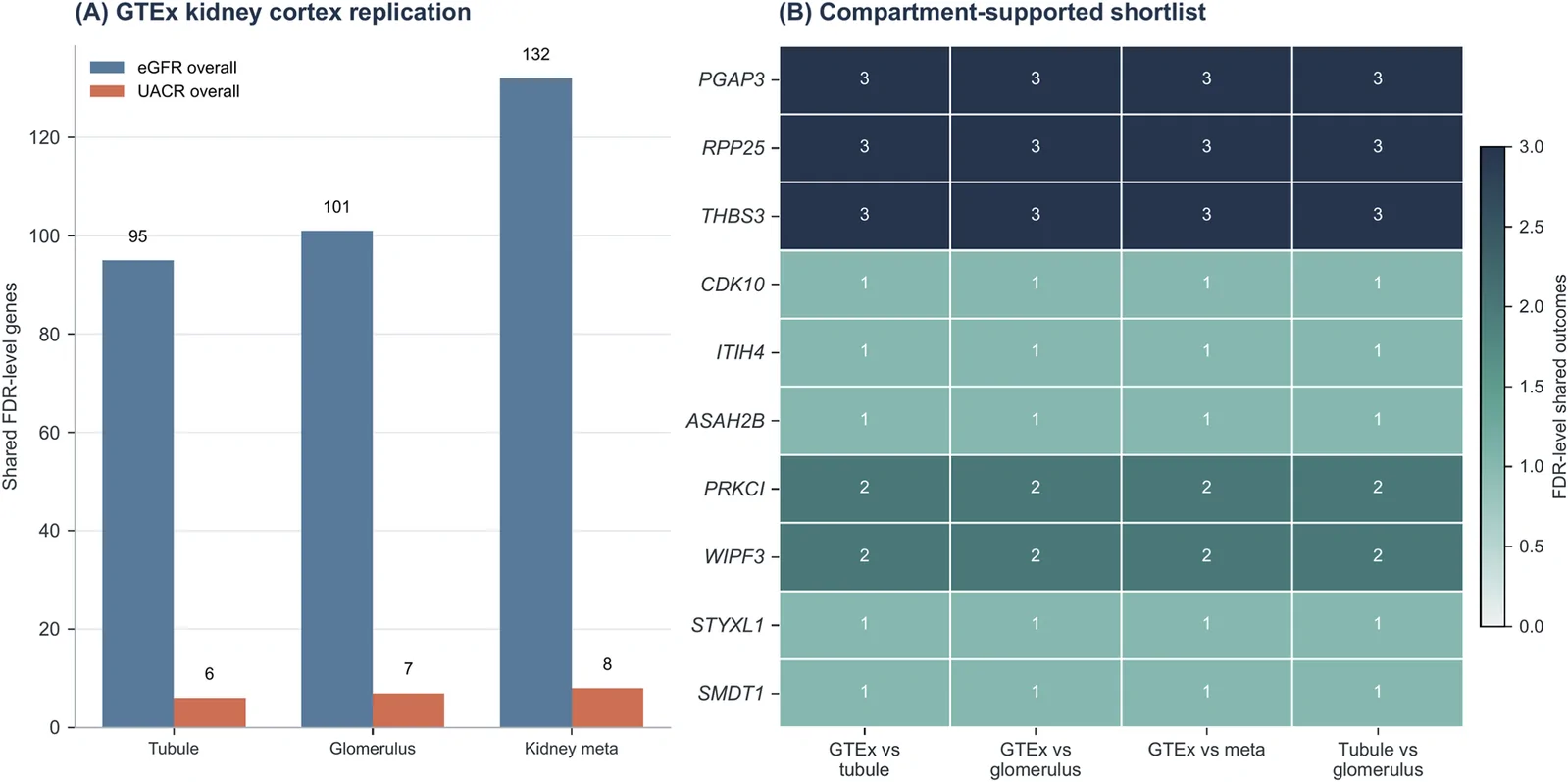
Kidney-compartment cross-resource reproducibility. **(A)** Counts of GTEx kidney-cortex FDR-level genes also observed at FDR levels in Susztak/NephQTL tubule, glomerulus, and kidney-meta resources for overall eGFR and overall UACR. **(B)** Heatmap of cross-resource reproducibility counts for the compartment-supported shortlist. These comparisons reuse the same kidney-trait outcome GWAS and therefore do not constitute independent external GWAS replication or a formal test of kidney specificity. GTEx, Genotype-Tissue Expression; FDR, false discovery rate; eGFR, estimated glomerular filtration rate; UACR, urinary albumin-to-creatinine ratio; QTL, quantitative trait locus.

### 3.3 Functional resources provided non-causal annotation for *PRKCI*

Protein and functional resources showed that *PRKCI* had the broadest annotation coverage within the panel. Among compartment-focused genes, nine had a detectable kidney protein signal in HPA and four had direct target-protein assay coverage in INTERVAL (Karlsson et al., 2021; Uhlen et al., 2015; Sun et al., 2018). *PRKCI* combined non-DKD Open Targets tractability annotations, druggable-family membership, kidney-IHC detection, plasma target-assay coverage, and direct target-pQTL evidence (Sun et al., 2018; Ochoa et al., 2023; Ghoussaini et al., 2021). *ITIH4* retained plasma target-assay coverage and antibody or GO-cellular-component annotation but lacked direct target-pQTL evidence; *WIPF3* support was driven mainly by kidney IHC. These are annotation and feasibility signals only. They do not demonstrate DKD therapeutic readiness, on-target efficacy, or safety (Supplementary Data S1; Figure 4).

**FIGURE 4:**
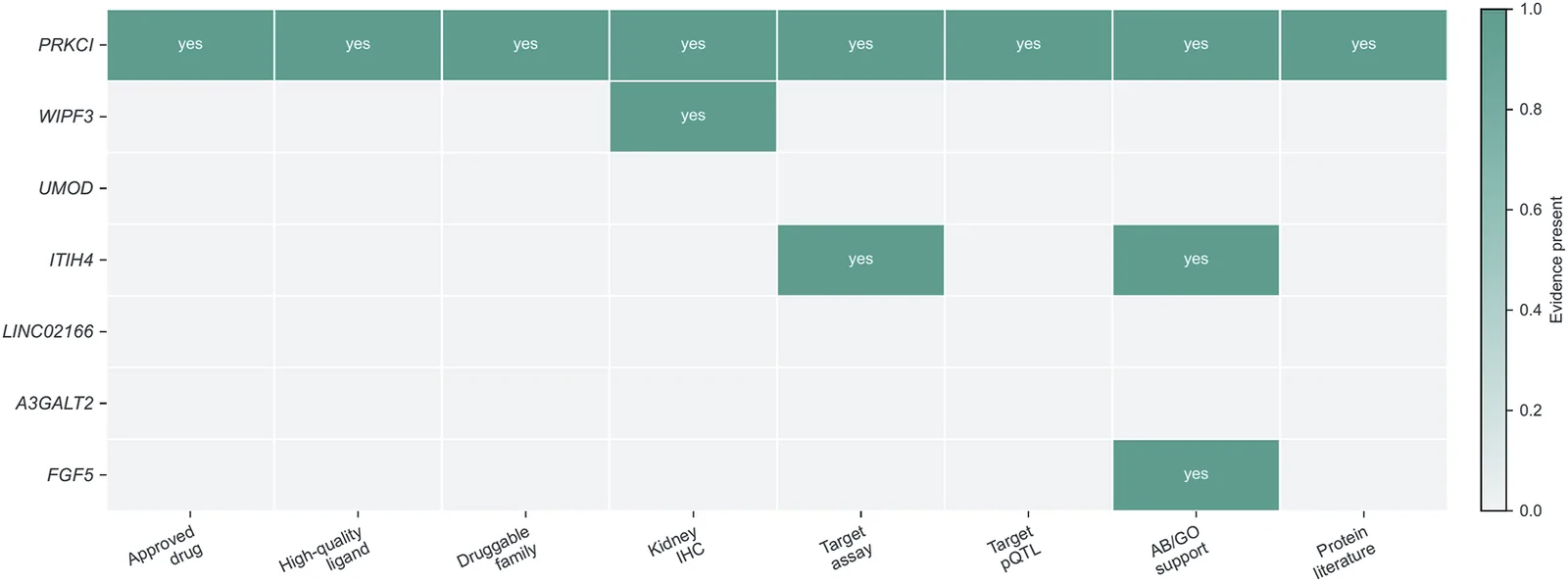
Functional and translational annotation evidence. Heatmap summarizing curated functional and translational annotations for the final seven-gene panel from Open Targets, HPA kidney-IHC, and INTERVAL target-assay/target-pQTL resources. Teal cells labeled “yes” indicate evidence present, and pale cells indicate no evidence in the queried resources. Abbreviations: HPA, Human Protein Atlas; IHC, immunohistochemistry; INTERVAL, the plasma proteome resource; pQTL, protein quantitative trait locus; GO, Gene Ontology; AB, antibody. Statistics: this panel is a binary evidence map from curated databases and the literature, so no inferential test is performed here.

### 3.4 Kidney single-cell analyses provided nephron and disease-state context

Healthy kidney single-cell annotation assigned nephron context to final-panel genes using cell-type expression resources (Karlsson et al., 2021). *PRKCI* showed the strongest expression in collecting duct cells with normalized protein-coding transcripts per million (pTPM) = 73.0. *UMOD* showed strong distal-tubular enrichment with pTPM = 883.8, consistent with thick ascending limb and distal nephron biology. *FGF5* showed proximal tubular enrichment with pTPM = 10.0, while *WIPF3* and *ITIH4* were detectable in proximal tubular cells.

Disease-state kidney single-cell analysis provided injured-cell context for selected genes using the primary disease atlas and a DKD-focused context sensitivity scan (Abedini et al., 2024; Tsai et al., 2023). In source-reported DKD-versus-control results, *UMOD* had differential-expression evidence across C_TAL, M_TAL, and iPT, while *PRKCI* had evidence in IC_A, IC_B, B_Naive, cDC, and Fibroblast_1. *UMOD* also had broader disease-state and CKD context, injured proximal-tubule trajectory membership (q = 6.02e-43), and fibrotic microenvironment membership. *ITIH4* appeared in the injured proximal-tubule trajectory analysis (q = 3.33e-10) and in all-disease differential-expression results for iPT and MyoFibVSMC. The secondary external DKD single-cell scan found only exploratory *UMOD* overlap and did not reproduce the full panel. Figure 5 now distinguishes source-reported differential-expression values, tested but non-significant cells, unavailable cells, binary source support, and absence from a source list; none of these categories are treated as causal or experimental validation.

**FIGURE 5:**
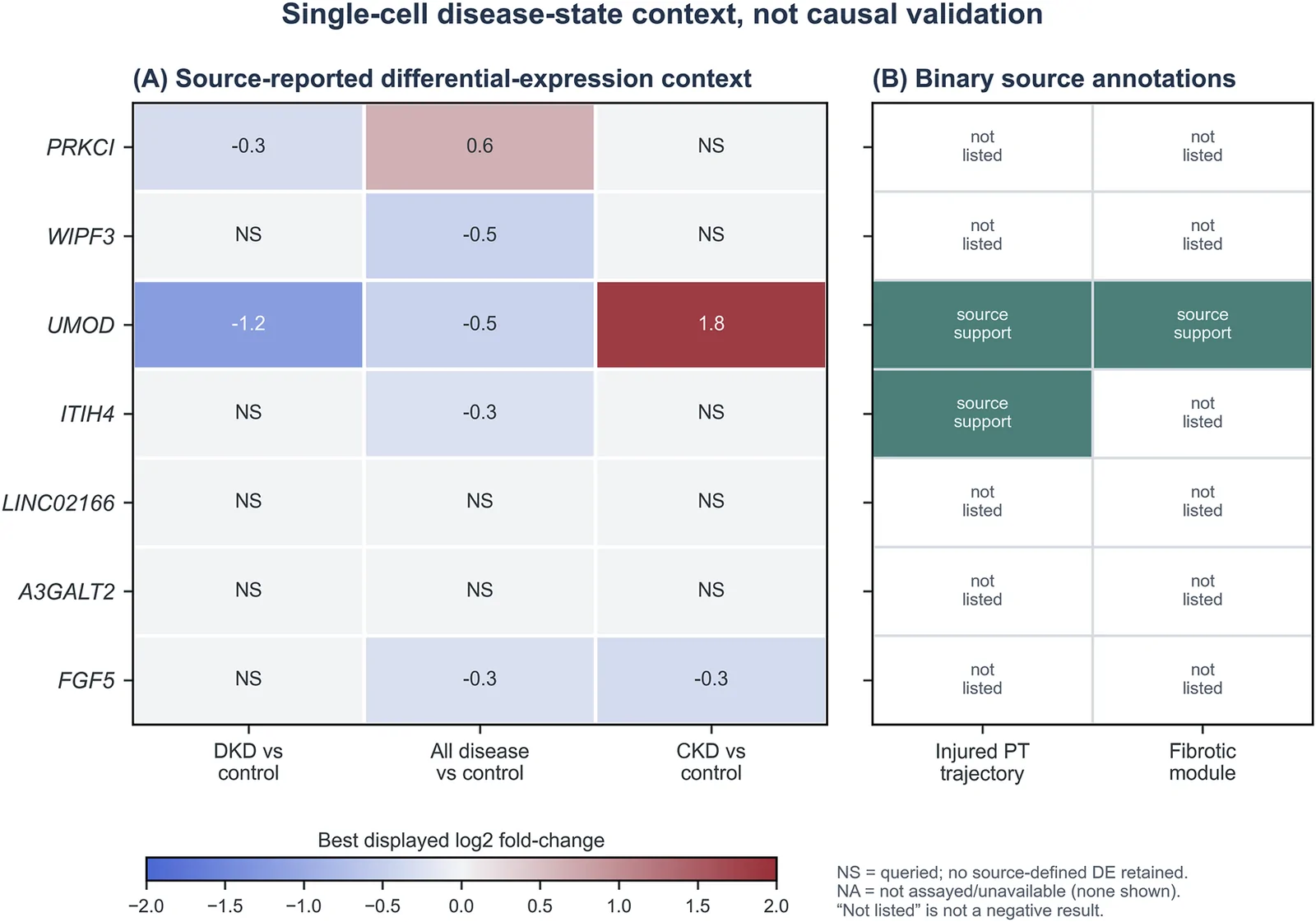
Single-cell disease-state context. Two-panel summary of source-reported single-cell disease-state context. **(A)** Disease-control columns display the best source-reported log2 fold change when at least one source-defined differential-expression cell type was retained. NS means that the gene was queried, but no such cell type was retained; NA means not assayed or unavailable (no NA cells occur in the displayed panel). **(B)** Trajectory/module columns distinguish binary source support from not listed; not listed is not a negative experimental result. These observational categories provide biological context, not causal or experimental validation. DKD, diabetic kidney disease; CKD, chronic kidney disease; PT, proximal tubule.

Independent disease-state expression context was assessed across two microdissected DKD compartment datasets and one kidney-biopsy RNA-seq dataset. *WIPF3*, *ITIH4*, *UMOD*, and *FGF5* had FDR-level differential-expression context in at least one disease-control contrast; *PRKCI* had nominal context only. *A3GALT2* and *LINC02166* were not represented in the selected processed external matrices. Assay coverage and tested but non-significant results are separated in Supplementary Data S9. Because directions were mixed and the analysis used processed observational matrices, these findings indicate disease-state perturbation context rather than independent causal or experimental validation.

### 3.5 Progression outcomes revealed a compartment-dominant *UMOD* axis

Progression analyses separated broad GTEx tissues from independent kidney compartments. Neither the GTEx kidney cortex nor whole blood produced FDR-significant genes for KP4CD diabetes-specific progression analyses. In contrast, kidney compartments identified recurrent screening signals centered on *UMOD*. *UMOD* was FDR-significant in tubule, glomerulus, and kidney meta for both general CKDGen progression endpoints, with the strongest kidney-meta result for ckdi25 (P = 2.08e-8, within-outcome FDR = 2.08e-4; study-wide BH-FDR = 3.42e-5) and a parallel rapid3 result (P = 5.12e-7, within-outcome FDR = 5.10e-3; study-wide BH-FDR = 4.56e-4) (Gorski et al., 2021). *UMOD* also met within-outcome FDR for KP4CD European (P = 3.01e-6, FDR = 0.0301; study-wide BH-FDR = 0.00177) and mixed-ancestry (P = 3.91e-6, FDR = 0.0395; study-wide BH-FDR = 0.00216) diabetes-specific eGFR decline, whereas the African-ancestry outcome was null (Robinson-Cohen et al., 2023). Because the exposure eQTLs were not ancestry matched, these cross-ancestry comparisons are exploratory. *FGF5* met within-outcome FDR for general CKDGen ckdi25 in the tubule and glomerulus but had no FDR-level diabetes-specific progression support. Thus, *UMOD* is the only final-panel gene with FDR-level evidence from diabetes-specific outcomes and general kidney traits; *FGF5* remains a secondary general-CKD progression signal (Supplementary Data S4, S5, S11, S12; Figure 6).

**FIGURE 6:**
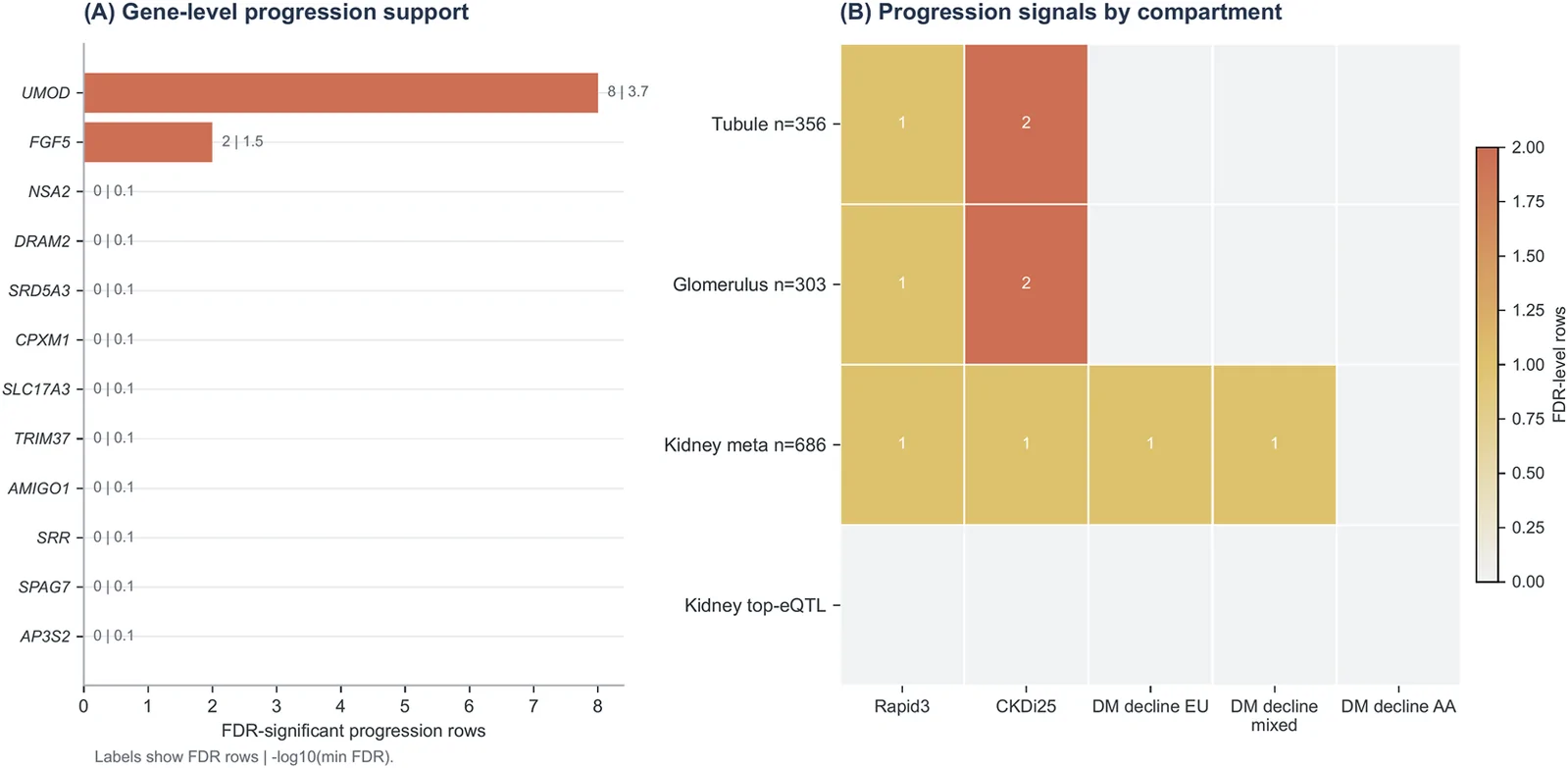
Progression signals across kidney compartments. **(A)** Gene-level progression support ranking based on the number of FDR-significant progression rows across CKDGen and KP4CD outcomes; the labels show “FDR rows | −log10 (min FDR)” for each gene. **(B)** Heatmap of progression signals by resource (tubule, glomerulus, kidney-meta, and kidney top-eQTL) and outcome (the source-defined CKDGen progression endpoints rapid3 and ckdi25 and the KP4CD diabetes-specific eGFR-decline analyses in European, mixed, and African ancestry). Row labels show the number of genes summarized where provided, and cell labels give the counts of FDR-level signals. Abbreviations: CKDGen, Chronic Kidney Disease Genetics consortium; KP4CD, Kidney Precision Medicine Project for Chronic Disease; FDR, false discovery rate; eGFR, estimated glomerular filtration rate; AA, African ancestry; EU, European ancestry. Statistics: counts are based on FDR significance within the progression scans, and the panel uses the minimum FDR per gene for the ranking summary.

### 3.6 Colocalization resolved a selective shared-variant subset

Single-signal Wakefield-ABF colocalization across 28 prioritized GTEx tissue-locus combinations identified seven default-prior tissue-level results with PP4 ≥ 0.80 (Giambartolomei et al., 2014; Wakefield, 2009). *PRKCI* and *WIPF3* had very high kidney-cortex shared-variant-compatible evidence for overall UACR in European ancestry (PP4 = 0.999947 and 0.999390) and remained robust across tested priors and windows (minimum prior PP4 = 0.994750 and 0.942453), with single-dominant-variant concentration proxies. In contrast, *LINC02166* (default PP4 = 0.979952; minimum prior PP4 = 0.328321), *ITIH4* (default PP4 = 0.935747/0.939024; minimum prior PP4 = 0.127121/0.133448), and *A3GALT2* (default PP4 = 0.912768/0.904391; minimum prior PP4 = 0.094725/0.086418) were prior-sensitive and had diffuse or multisignal proxy patterns. Therefore, these three genes were downgraded to sensitivity-limited secondary loci. *PGAP3*, *CDK10*, *RPP25*, and *SMDT1* showed PP3-dominant or weak patterns and were not retained. Figure 7 now shows default PP4, minimum PP4 across tested priors, and the robustness flag. No result is described as proof of a single causal variant, and the sensitivity proxies do not replace LD-aware conditional/SuSiE colocalization (Supplementary Data S6, S10; Figure 7; Wallace, 2021).

**FIGURE 7:**
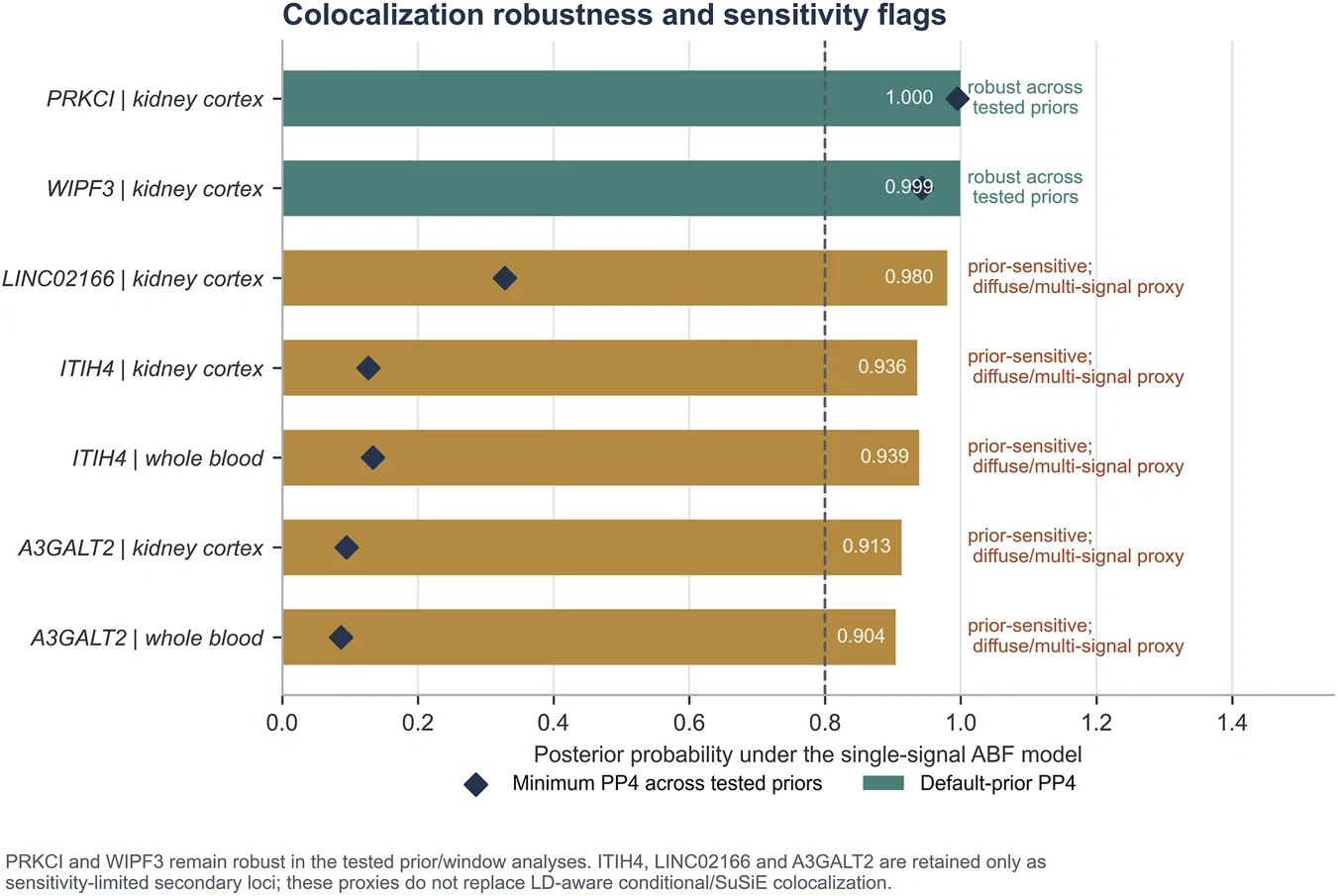
Colocalization evidence for prioritized loci. Default-prior and sensitivity summary for the five final-panel genes with default PP4 ≥ 0.80 in the GTEx kidney cortex or whole blood. Horizontal bars show default-prior PP4; diamonds show the minimum PP4 across tested shared-variant priors; teal identifies *PRKCI* and *WIPF3* as robust across the tested prior/window checks with single-dominant-variant concentration proxies; ochre and text flags identify *ITIH4*, *LINC02166*, and *A3GALT2* as prior-sensitive with diffuse/multisignal proxies. The dashed line marks PP4 = 0.80. These are single-signal ABF results and sensitivity proxies, not LD-aware conditional or SuSiE colocalization and not proof of one causal variant. ABF, approximate Bayes factor; PP4, posterior probability of shared-variant-compatible evidence.

### 3.7 Integrated prioritization supports a seven-gene candidate panel

The manuscript-facing panel contains seven follow-up genes, with outcome scope and limitations shown explicitly. *PRKCI* and *WIPF3* are primary albuminuria follow-up loci supported by general overall-UACR outcomes and robust single-signal ABF colocalization; neither has FDR-level diabetes-specific UACR support. *ITIH4* and *LINC02166* are secondary general-eGFR loci because their colocalization is prior-sensitive and diffuse, despite cross-resource screening support. *A3GALT2* is a secondary general-UACR locus for the same reason and has limited orthogonal annotation. *UMOD* is the primary progression follow-up gene because it combines diabetes-specific European/mixed-ancestry progression screening with general kidney-trait and compartment context. *FGF5* is a secondary general-CKD progression gene without FDR-level diabetes-specific progression evidence. Table 1 uses neutral primary/secondary panel labels rather than clinical-sounding tiers; full instrument and layer-by-layer traceability is provided in Supplementary Data S11.

## 4 Discussion

The principal result is a scope-qualified follow-up panel rather than a set of established DKD-causal genes. Kidney-compartment eQTL resources add biological localization and cross-resource recurrence beyond bulk GTEx tissues (GTEx Consortium, 2020; GTEx Consortium et al., 2017; Qiu et al., 2018), but reuse of the same outcome GWAS means that compartment recurrence is not independent external GWAS replication. Diabetes-specific progression results are most informative for *UMOD*; most other final-panel support comes from overall eGFR, overall UACR, or general CKD progression. Disease-state single-cell, external expression, protein, and tractability layers help describe plausible tissue and feasibility context but cannot validate a causal mechanism. Colocalization further separates robust shared-variant-compatible loci from sensitivity-limited local patterns under the single-signal ABF model (Giambartolomei et al., 2014; Wakefield, 2009; Wallace, 2021).

Within the albuminuria axis, *PRKCI* and *WIPF3* have robust single-signal ABF colocalization with overall UACR and recurrent kidney-compartment screening support. *PRKCI* additionally has kidney protein, plasma target-pQTL, collecting-duct, disease-state, and non-DKD tractability annotations (Karlsson et al., 2021; Sun et al., 2018; Ochoa et al., 2023; Ghoussaini et al., 2021). These features make *PRKCI* the most extensively annotated albuminuria follow-up locus in this study, not a leading causal DKD gene or a DKD-ready therapeutic target. *WIPF3* has less translational infrastructure. *A3GALT2* remains secondary because its PP4 is strongly prior-sensitive, its SNP-level support is diffuse, and its biological annotation is limited.

The eGFR axis is based on general kidney-function outcomes rather than diabetes-specific evidence. *ITIH4* has cross-tissue and kidney-compartment screening support, proximal-tubule and injured-PT context, and intermediate protein annotation, but kidney-cortex and whole-blood colocalization is prior-sensitive and diffuse. *LINC02166* has high default kidney-cortex PP4 but lower minimum PP4 under prior sensitivity, a diffuse local pattern, and little orthogonal annotation. Neither locus should be described as kidney specific, DKD specific, or robustly resolved to one causal variant.

The progression axis provides the clearest diabetes-specific contribution and also exposes ancestry limitations. *UMOD* recurred across general CKDGen progression outcomes, kidney compartments, diabetic eGFR status, and KP4CD European/mixed-ancestry diabetes-specific eGFR-decline outcomes (Gorski et al., 2021; Robinson-Cohen et al., 2023). The smaller African-ancestry outcome was null, and European-derived exposure eQTLs are not ancestry matched; therefore, LD and allele-frequency mismatch preclude an ancestry-general interpretation (Mägi et al., 2017). *FGF5* is supported by general ckdi25 and overall-eGFR results but not by diabetes-specific progression at FDR, so it remains secondary.

The disease-state analyses place selected loci in injured or diseased kidney contexts (Abedini et al., 2024; Lake et al., 2023; Tsai et al., 2023; Woroniecka et al., 2011; Fan et al., 2019). *UMOD* had the broadest single-cell disease-state context, *PRKCI* had disease-state context in addition to healthy collecting-duct expression, and *ITIH4* appeared in an injured proximal-tubule trajectory. External processed expression matrices provided targeted perturbation context for several genes. These observational layers are neither independent genetic replication nor biological validation, and a missing assay or absent source-list entry is not a negative experimental result.

The study uses explicit downgrade rules rather than carrying every statistically attractive screening result forward. *PGAP3*, *CDK10*, *RPP25*, and *SMDT1* were excluded after PP3-dominant or weak colocalization. *ITIH4*, *LINC02166*, and *A3GALT2* were retained only as secondary loci after prior sensitivity and diffuse-signal review. *PRKCI* and *WIPF3* remained primary albuminuria follow-up loci because their single-signal ABF results were robust across the tested specifications, while *UMOD* was prioritized separately on the progression axis. The stagewise universe, FDR families, thresholds, representative instruments, retained candidates, and downgrade rules are auditable in Supplementary Data S11 and S12.

Six MR-specific limitations warrant particular emphasis. First, the relevance assumption was screened using significant cis-eQTLs and F statistic > 10, but the independence and exclusion-restriction assumptions cannot be established from these summary data. Cis restriction, harmonization, and colocalization reduce some ambiguity but do not rule out confounding pathways, horizontal pleiotropy, or reverse direction; Steiger filtering was not performed (Skrivankova et al., 2021; Burgess et al., 2023; Hemani et al., 2017). Second, no formal *a priori* power calculation was performed, and statistical power differs substantially across exposure and outcome resources. Small GTEx kidney-cortex and diabetes-specific or African-ancestry outcome samples are especially vulnerable to imprecision, so null or non-retained results should not be interpreted as evidence of no effect. Third, F statistic > 10 reduces but does not eliminate weak-instrument bias. Selecting significant eQTLs and the best instrument may introduce winner’s-curse bias and overstate apparent strength, and unknown participant overlap means that the direction and magnitude of residual bias cannot be specified confidently (Jiang et al., 2023). Fourth, using one representative cis SNP avoids combining correlated instruments without a matched-LD reference, but residual LD with variants affecting other genes or pathways can violate the instrumental-variable assumptions. The single-signal ABF colocalization model does not prove the absence of multiple causal signals. Fifth, one instrument cannot characterize genetic heterogeneity across regulatory variants or variant-specific mechanisms and precludes Cochran’s Q, I-squared, MR-Egger, weighted-median, and leave-one-out analyses. Sixth, the data sources impose additional constraints: heterogeneous endpoint definitions and original covariate adjustments, inability to enforce identical covariate sets, unavailable participant-overlap counts, incomplete variance information, predominantly European and non-diabetic eQTL resources, ancestry mismatch for African-ancestry outcomes, and lack of independent external kidney-GWAS replication.

Additional limitations apply to the broader prioritization framework. Primary BH families were outcome/resource specific; the added 597,099-row study-wide BH analysis is a sensitivity check across correlated and partly repeated branches, not a replacement for the prespecified families. Kidney-compartment recurrence reuses outcome GWAS and does not establish kidney specificity; no formal cross-tissue specificity test was performed. Disease-state single-cell, external expression, protein, and tractability resources provide observational context only and cannot replace perturbational validation. Colocalization was limited to dense GTEx locus data: no Susztak/NephQTL colocalization, matched-LD conditional analysis, or SuSiE analysis were performed, and prior/window or concentration proxies only flag sensitivity (Wallace, 2021). Finally, lifelong cis-eQTL effects should not be equated directly with acute pharmacologic or therapeutic modification. These constraints motivate ancestry-matched eQTL/GWAS replication, LD-aware multisignal colocalization, and targeted perturbation in diabetes-relevant kidney models.

## 5 Conclusion

This exploratory public-data framework identifies a scope-qualified follow-up panel rather than definitive causal DKD genes. *PRKCI* and *WIPF3* are robustly colocalized general-UACR loci; *ITIH4*, *LINC02166*, and *A3GALT2* are sensitivity-limited secondary general-trait loci; *UMOD* is the only final-panel gene with FDR-level diabetes-specific and general kidney-trait support; and *FGF5* remains a secondary general-progression signal. Independent ancestry-matched replication, LD-aware multisignal colocalization, and experimental validation are required before causal, DKD-specific, or therapeutic claims.

## Data Availability

Processed result tables, figure files and source data, analysis scripts, configuration files, environment documentation, and provenance notes are directly accessible in the publicly released revised Zenodo record https://doi.org/10.5281/zenodo.21670092 (Chen and Bi, 2026; published and verified accessible 29 July 2026; concept DOI https://doi.org/10.5281/zenodo.20698301). The record contains the primary archive project01_dkd_causal_ atlas_zenodo_generic_upload_20260615.zip (160,806,695 bytes; MD5 48c40ce5663aeb2a4bb7378ffc53d7a1) and the revision addendum Zenodo_revision_addendum_2026‐07‐29.zip (2,344,948 bytes; MD5 1702f7ab12c97300513707f1bef066e8). The primary archive includes environment.yml, config/datasets.tsv, metadata/file_manifest_sha256.tsv, metadata/reproducibility_notes.md, processed tables, figure source data, and scripts; the addendum contains Supplementary Data S11 and S12, revised Figures 2/5/7, the revision script, environment file, SHA‐ 256 manifest, and Reproducibility Quickstart. Raw third‐party GWAS, eQTL, single‐cell, protein, pathway, and annotation source files are not redistributed and must be obtained from original providers under their access conditions. No individual‐ level participant data, controlled‐access source files, journal administrative files, or author correspondence are included.

## Glossary

AA: African ancestry
ABF: Approximate Bayes factor
B_Naive: Naive B cell
cDC: Conventional dendritic cell
CKD: Chronic kidney disease
CKDGen: Chronic Kidney Disease Genetics consortium
C_TAL: Cortical thick ascending limb
DKD: Diabetic kidney disease
DM: Diabetes mellitus
EA: European ancestry
eGFR: Estimated glomerular filtration rate
eQTL: Expression quantitative trait locus
FDR: False discovery rate
GO: Gene Ontology
GRCh37: Genome Reference Consortium Human Build 37
GTEx: Genotype-Tissue Expression project
GWAS: Genome-wide association study
HPA: Human Protein Atlas
IC_A: Type A intercalated cell
IC_B: Type B intercalated cell
IHC: Immunohistochemistry
iPT: Injured proximal tubule
KP4CD: Kidney Precision Medicine Project for Chronic Disease
IV: Instrumental variable
MR: Mendelian randomization
M_TAL: Medullary thick ascending limb
MyoFibVSMC: Myofibroblast/vascular smooth muscle cell cluster
PP3: Posterior probability supporting distinct association signals in colocalization
PP4: Posterior probability for a shared variant under the ABF colocalization model
pQTL: Protein quantitative trait locus
pTPM: Protein-coding transcripts per million
SNP: Single-nucleotide polymorphism
UACR: Urinary albumin-to-creatinine ratio
