# Supplementary material for: Kidney-compartment genomic prioritization across diabetic and population kidney traits: an exploratory Mendelian randomization and colocalization study
Source: Front Genet. 2026 Oct 28;17:1921914. doi: 10.3389/fgene.2026.1921914 (PMC13752862; doi:10.3389/fgene.2026.1921914)
Supplement: Supplementary file 1 [file DataSheet1.pdf]

REPRODUCIBILITY QUICKSTART

Processed data, source audit, and manuscript-figure regeneration

Project: Kidney-compartment genomic prioritization across diabetic and population kidney traits
Public revised record: https://doi.org/10.5281/zenodo.21670092
Concept DOI: https://doi.org/10.5281/zenodo.20698301
Verified: Published and publicly accessible on 29 July 2026
Primary archive: project01_dkd_causal_atlas_zenodo_generic_upload_20260615.zip
Primary archive checksum: MD5 48c40ce5663aeb2a4bb7378ffc53d7a1
Revision addendum: Zenodo_revision_addendum_2026-07-29.zip
Revision addendum checksum: MD5 1702f7ab12c97300513707f1bef066e8

1. What the archive contains

The Zenodo archive is a processed-data reproducibility package. It contains scripts, configuration, environment definitions, result tables, figure source data, generated figures, provenance metadata, and manuscript-context files. It deliberately does not redistribute large or controlled third-party raw GWAS, eQTL, single-cell, protein, or annotation datasets.

Environment: environment.yml

Dataset/provenance registry: config/datasets.tsv

Per-file SHA-256 manifest: metadata/file_manifest_sha256.tsv

Archive notes: metadata/reproducibility_notes.md

Analysis code: scripts/

Processed outputs: results/tables/ and results/figures/

Manuscript-facing copies: manuscript_context/

2. Download and verify the Zenodo archive

PowerShell:

```
$url =
"https://zenodo.org/records/21670092/files/project01_dkd_causal_atlas_zenodo_generic_upload_
20260615.zip?download=1"
```

```
Invoke-WebRequest -Uri $url -OutFile
"project01_dkd_causal_atlas_zenodo_generic_upload_20260615.zip"
Get-FileHash "project01_dkd_causal_atlas_zenodo_generic_upload_20260615.zip" -Algorithm MD5
```

Expected MD5: 48c40ce5663aeb2a4bb7378ffc53d7a1.

Linux/macOS:

```
curl -L -o project01_dkd_causal_atlas_zenodo_generic_upload_20260615.zip \

"https://zenodo.org/records/21670092/files/project01_dkd_causal_atlas_zenodo_generic_upload_
20260615.zip?download=1"
md5sum project01_dkd_causal_atlas_zenodo_generic_upload_20260615.zip
```

3. Create the software environment

```
unzip project01_dkd_causal_atlas_zenodo_generic_upload_20260615.zip
cd zenodo_generic_upload_20260615
conda env create -f environment.yml
conda activate dkd-causal-atlas
```

The environment file specifies Python 3.10, R 4.3, pandas, PyYAML, requests, pyarrow, matplotlib, JupyterLab, data.table, optparse, devtools, and supporting packages.

4. Audit source links

The included source checker reads the public URL registry and writes HTTP status, final URL, content type, response length, timing, and error fields.

```
python scripts/check_sources.py \
  --manifest config/datasets.tsv \
  --output results/reproducibility/source_url_audit.tsv \
  --timeout 20
```

5. Regenerate processed manuscript figures

The following command uses included processed result/source-data tables and does not require redistribution of the full third-party raw datasets:

```
python scripts/make_genome_medicine_submission_figures.py
```

Expected output directory: results/figures/genome_medicine_submission/. The revision package separately supplies revised Figures 2, 5, and 7 and the corresponding revision script prepare_frontiers_major_revision_20260729.py.

6. Inspect checksums inside the archive

metadata/file_manifest_sha256.tsv contains path, byte count, and SHA-256 for each packaged file. A minimal cross-platform verification snippet is:

```
python - <<'PY'
import csv, hashlib, pathlib
root = pathlib.Path('.')
with open(root/'metadata/file_manifest_sha256.tsv', encoding='utf-8') as handle:
    for row in csv.DictReader(handle, delimiter='\t'):
        path = root / row['path']
        digest = hashlib.sha256(path.read_bytes()).hexdigest()
        assert digest == row['sha256'], row['path']
print('All packaged SHA-256 values match.')
PY
```

On Windows PowerShell, the same table can be inspected with Import-Csv -Delimiter "`t" and files can be checked with Get-FileHash -Algorithm SHA256.

7. Full-rerun boundary

A full end-to-end rerun requires downloading the public raw sources listed in config/datasets.tsv and observing each provider's access terms. Large or controlled third-party inputs are not redistributed in Zenodo. The public package supports independent inspection of provenance, code, processed tables, figure source data, and manuscript-facing outputs; it should not be interpreted as including every raw source file.

Likewise, the study is an exploratory candidate-prioritization analysis. The archive does not provide experimental validation, independent external kidney-GWAS replication, ancestry-matched eQTL resources for every outcome, or LD-aware conditional/SuSiE colocalization.

8. Revision-package additions

Zenodo_revision_addendum_2026-07-29.zip in the revised record contains Supplementary Data S11/S12, revised Figures 2/5/7, the revision script, environment file, SHA-256 manifest, and this quickstart.
