# Supplementary material for: Kidney-compartment genomic prioritization across diabetic and population kidney traits: an exploratory Mendelian randomization and colocalization study
Source: Front Genet. 2026 Oct 28;17:1921914. doi: 10.3389/fgene.2026.1921914 (PMC13752862; doi:10.3389/fgene.2026.1921914)
Supplement: Supplementary file 2 [file DataSheet3.pdf]

Supplementary Material Manifest

Target journal: Frontiers in Genetics; article type: Original Research.

This revised manifest lists supplementary files prepared for the Frontiers in Genetics major-revision package. Each file is supplied separately as editable source data, a reviewer-facing reproducibility guide, or a standalone MR reporting summary.

File label	Title	Description
Supplementary Data S1	Functional and translational support	Open Targets, HPA, and INTERVAL-derived tractability and functional support for the final seven-gene panel.
Supplementary Data S2	Disease-state kidney single-cell support	Final-panel gene support from DKD versus control, all disease versus control, CKD versus control, injured proximal tubule trajectory, and fibrotic microenvironment resources.
Supplementary Data S3	Secondary disease-state scan summary	Context sensitivity scan of a second external DKD single-cell supplement for final-panel gene overlap; absence from a source list is not treated as a negative result.
Supplementary Data S4	Progression summary	Resource-level results for CKDGen and KP4CD progression outcomes across GTEx and kidney-compartment resources.
Supplementary Data S5	Progression gene summary	Gene-level summary of recurrent and FDR-significant progression support.
Supplementary Data S6	Gene-level colocalization summary	Maximum PP4 and colocalization class for prioritized genes across tested tissues and outcomes.
Supplementary Data S7	Integrated seven-gene priority panel	Manuscript-facing Table 1 source data and supporting-evidence workbook for disease axis, progression, colocalization, cell-state and functional layers.
Supplementary Data S8	MR safeguards and interpretation limits	Reporting-oriented safeguards for best-instrument MR, sensitivity interpretation, instrument strength, and claim boundaries.
Supplementary Data S9	Independent DKD disease-state expression context	Targeted expression-context assessment of final-panel genes across GSE30528, GSE30529, and GSE142025, including dataset metadata, assay coverage, differential-expression summaries, gene-level context summary, and methods notes; this is not causal validation.
Supplementary Data S10	Colocalization and specificity checks	Prior sensitivity, window-level checks, and supporting evidence for tissue-locus colocalization interpretation.
MR reporting summary	MR reporting summary	Standalone MR reporting summary for rapid editorial/statistical review. The same summary is also included in Supplementary Data S8 so that MR safeguards, data-source access information, and reporting notes remain together in one transparency workbook.

Major-revision additions

File label	Title	Description
Supplementary Data S11	Final-candidate evidence scope and instruments	Diabetes-specific versus general kidney-trait evidence map; representative SNP, F statistic, exposure/outcome beta and SE, harmonization direction, MR estimate, P value, within-outcome/branch/study-wide FDR, outcome source, selection path, and downgrade rules.
Supplementary Data S12	Study-wide FDR sensitivity	BH-FDR sensitivity across all 597,099 MR screening rows, including universe counts, resource-outcome summaries, and final-panel rows. Correlated and repeated branches are explicitly flagged.
Reproducibility Quickstart	Reviewer-facing reproducibility and archive guide	Direct Zenodo DOI, archive filename and checksum, file-manifest and environment locations, minimal verification commands, package file map, and explicit limits on redistribution and full raw-data reruns.
