# Supplementary material for: Kidney-compartment genomic prioritization across diabetic and population kidney traits: an exploratory Mendelian randomization and colocalization study
Source: Front Genet. 2026 Oct 28;17:1921914. doi: 10.3389/fgene.2026.1921914 (PMC13752862; doi:10.3389/fgene.2026.1921914)
Supplement: Supplementary file 3 [file Table13.docx]

**STROBE-MR checklist of recommended items to address in reports of Mendelian randomization studies**^1^ ^2^

Completed for: Kidney-compartment genomic prioritization across diabetic and population kidney traits: an exploratory Mendelian randomization and colocalization study | Frontiers in Genetics | 13 August 2026

| **Item No.** | **Section** | **Checklist item** | **Page No.** | **Relevant text from manuscript** |
| --- | --- | --- | --- | --- |
| 1 | **TITLE and ABSTRACT** | Indicate Mendelian randomization (MR) as the study’s design in the title and/or the abstract if that is a main purpose of the study | 1–2 | Title and Abstract identify exploratory Mendelian randomization and single-SNP Wald-ratio screening. |
|  | **INTRODUCTION** |  |  |  |
| 2 | **Background** | Explain the scientific background and rationale for the reported study. What is the exposure? Is a potential causal relationship between exposure and outcome plausible? Justify why MR is a helpful method to address the study question | 2–3 | Introduction explains the gene-expression exposures, kidney outcomes, rationale for MR screening, and need for colocalization. |
| 3 | **Objectives** | State specific objectives clearly, including pre-specified causal hypotheses (if any). State that MR is a method that, under specific assumptions, intends to estimate causal effects | 3 | Objectives distinguish screening from causal interpretation and state that causal interpretation requires the IV assumptions. |
|  | **METHODS** |  |  |  |
| 4 | **Study design and data sources** | Present key elements of the study design early in the article. Consider including a table listing sources of data for all phases of the study. For each data source contributing to the analysis, describe the following: | 3–8 | Study design and all data sources are described in Materials and Methods; Figure 1 summarizes the workflow. |
|  | a) | Setting: Describe the study design and the underlying population, if possible. Describe the setting, locations, and relevant dates, including periods of recruitment, exposure, follow-up, and data collection, when available. | 3–4 | Public GTEx, Susztak/NephQTL, CKDGen, KP4CD, and contextual resources and their source populations are described and cited. |
|  | b) | Participants: Give the eligibility criteria, and the sources and methods of selection of participants. Report the sample size, and whether any power or sample size calculations were carried out prior to the main analysis | 4–5 | Available study sample sizes are reported. No formal a priori power calculation was performed; this is stated explicitly. |
|  | c) | Describe measurement, quality control and selection of genetic variants | 4–5 | Variant selection/QC includes liftOver, allele checks, minor allele count, cis-eQTL significance, F statistic >10, harmonization, and palindromic-SNP exclusion. |
|  | d) | For each exposure, outcome, and other relevant variables, describe methods of assessment and diagnostic criteria for diseases | 4 | Exposure resources and eleven eGFR, UACR, and progression outcomes are defined with source citations and sample sizes. |
|  | e) | Provide details of ethics committee approval and participant informed consent, if relevant | 8, 14 | Secondary analysis of de-identified summary data; original approvals/consent are in source reports. No new participants or specimens. |
| 5 | **Assumptions** | Explicitly state the three core IV assumptions for the main analysis (relevance, independence and exclusion restriction) as well assumptions for any additional or sensitivity analysis | 5 | Relevance, independence, and exclusion restriction are stated explicitly, with what could and could not be assessed. |
| 6 | **Statistical methods: main analysis** | Describe statistical methods and statistics used | 4–7 | Single-SNP Wald ratio, delta-method SE, harmonization, multiple testing, and colocalization methods are described. |
|  | a) | Describe how quantitative variables were handled in the analyses (i.e., scale, units, model) | 5 | Expression and outcome effects are used on the source-reported quantitative/log-odds scales; direction follows harmonized alleles. |
|  | b) | Describe how genetic variants were handled in the analyses and, if applicable, how their weights were selected | 4–5 | One representative cis variant is selected by exposure P value, F statistic, and distance to transcription start site; F >10 is required. |
|  | c) | Describe the MR estimator (e.g. two-stage least squares, Wald ratio) and related statistics. Detail the included covariates and, in case of two-sample MR, whether the same covariate set was used for adjustment in the two samples | 5 | Wald-ratio estimator and uncertainty are defined. Original covariate adjustments were inherited; identical covariate sets could not be enforced. |
|  | d) | Explain how missing data were addressed | 5 | Rows lacking required allele/effect/SE or a matched variant were excluded; no individual-level imputation was performed. |
|  | e) | If applicable, indicate how multiple testing was addressed | 5, 7–8 | Primary BH-FDR families and the study-wide 597,099-row BH sensitivity analysis are described. |
| 7 | **Assessment of assumptions** | Describe any methods or prior knowledge used to assess the assumptions or justify their validity | 5, 12–13 | Relevance was screened with significant cis-eQTLs/F >10; limits in assessing independence and exclusion restriction are explicit. |
| 8 | **Sensitivity analyses and additional analyses** | Describe any sensitivity analyses or additional analyses performed (e.g. comparison of effect estimates from different approaches, independent replication, bias analytic techniques, validation of instruments, simulations) | 5, 7, 10–11 | Study-wide FDR and colocalization prior/window/concentration checks are described; unavailable multi-instrument and SuSiE analyses are stated. |
| 9 | **Software and pre-registration** |  |  |  |
|  | a) | Name statistical software and package(s), including version and settings used | 7, 15 | Python 3.10 and R 4.3 are stated; full package versions/settings are archived in environment.yml and configuration files. |
|  | b) | State whether the study protocol and details were pre-registered (as well as when and where) | 3 | The exploratory workflow was not preregistered; post-review additions are labelled transparently. |
|  | **RESULTS** |  |  |  |
| 10 | **Descriptive data** |  | 8–11 | Results report the screened universes, retained counts, candidate flow, and final panel; Figure 1 summarizes the workflow. |
|  | a) | Report the numbers of individuals at each stage of included studies and reasons for exclusion. Consider use of a flow diagram | 4, 8 | Source study sample sizes and analysis-stage retained counts are reported; original participant selection is cited to source studies. |
|  | b) | Report summary statistics for phenotypic exposure(s), outcome(s), and other relevant variables (e.g. means, SDs, proportions) | 4, 8–11 | Outcome sample sizes and genetic association summaries are reported; individual-level phenotype summaries were not available for this summary-data study. |
|  | c) | If the data sources include meta-analyses of previous studies, provide the assessments of heterogeneity across these studies | 4; source reports | The manuscript uses published meta-analysis summary statistics; original-study heterogeneity assessments remain in the cited source reports and were not re-estimated. |
|  | d) | For two-sample MR:  i.  Provide justification of the similarity of the genetic variant-exposure associations between the exposure and outcome samples  ii.  Provide information on the number of individuals who overlap between the exposure and outcome studies | 4–5, 13 | Exposure/outcome ancestry similarity is discussed qualitatively. Participant overlap could not be quantified and was not assumed absent. |
| 11 | **Main results** |  | 8–11 | Main screening and colocalization results are reported by outcome scope, resource, and gene. |
|  | a) | Report the associations between genetic variant and exposure, and between genetic variant and outcome, preferably on an interpretable scale | 8–11; S11–S12 | Representative variant–exposure and variant–outcome beta/SE values, alleles, F statistics, and sources are in S11/S12. |
|  | b) | Report MR estimates of the relationship between exposure and outcome, and the measures of uncertainty from the MR analysis, on an interpretable scale, such as odds ratio or relative risk per SD difference | 8–11; S11–S12 | Wald-ratio beta/SE/CI/P/FDR are reported as screening estimates on source-defined expression and outcome scales. |
|  | c) | If relevant, consider translating estimates of relative risk into absolute risk for a meaningful time period | Not applicable | Absolute-risk translation is not appropriate for heterogeneous gene-expression screening estimates and was not performed. |
|  | d) | Consider plots to visualize results (e.g. forest plot, scatterplot of associations between genetic variants and outcome versus between genetic variants and exposure) | Figs 2, 6–7; 21–23 | Heatmaps and colocalization displays visualize screening direction, progression support, and PP4 sensitivity. |
| 12 | **Assessment of assumptions** |  | 8–13 | Instrument-strength and assumption-related limitations are reported with explicit interpretation boundaries. |
|  | a) | Report the assessment of the validity of the assumptions | 5, 12–13 | Relevance screen and the inability to establish independence/exclusion restriction are reported. |
|  | b) | Report any additional statistics (e.g., assessments of heterogeneity across genetic variants, such as *I^2^*, Q statistic or E-value) | Not applicable | One representative instrument precludes I-squared, Cochran's Q, MR-Egger, and other multi-instrument statistics. |
| 13 | **Sensitivity analyses and additional analyses** |  | 8–11; S10–S12 | Sensitivity and additional analyses are reported in Results and Supplementary Data S10–S12. |
|  | a) | Report any sensitivity analyses to assess the robustness of the main results to violations of the assumptions | 10–11; S10 | Colocalization prior/window/concentration sensitivity results are reported and explicitly do not replace LD-aware analyses. |
|  | b) | Report results from other sensitivity analyses or additional analyses | 8–11; S11–S12 | Study-wide BH-FDR, evidence-scope mapping, representative instruments, and downgrade rules are reported. |
|  | c) | Report any assessment of direction of causal relationship (e.g., bidirectional MR) | Not performed | Steiger filtering and bidirectional MR were not performed because scale/variance information was incomplete; reverse direction remains unresolved. |
|  | d) | When relevant, report and compare with estimates from non-MR analyses | 9–12 | Single-cell, expression, protein, and tractability layers are reported separately as biological context, not validation or comparable causal estimates. |
|  | e) | Consider additional plots to visualize results (e.g., leave-one-out analyses) | Not applicable | Leave-one-out plots are not possible for a one-instrument design. |
|  | **DISCUSSION** |  |  |  |
| 14 | **Key results** | Summarize key results with reference to study objectives | 11 | Discussion opens with the scope-qualified seven-gene result and relates it to the study objective. |
| 15 | **Limitations** | Discuss limitations of the study, taking into account the validity of the IV assumptions, other sources of potential bias, and imprecision. Discuss both direction and magnitude of any potential bias and any efforts to address them | 12–13 | Six MR-specific limitations address assumptions, power, weak instruments, LD, genetic heterogeneity, and data limitations, including possible bias. |
| 16 | **Interpretation** |  | 11–13 | Discussion and Conclusions provide cautious interpretation and avoid definitive causal claims. |
|  | a) | Meaning: Give a cautious overall interpretation of results in the context of their limitations and in comparison with other studies | 11–13 | Results are interpreted as hypothesis-generating and in context of published kidney resources. |
|  | b) | Mechanism: Discuss underlying biological mechanisms that could drive a potential causal relationship between the investigated exposure and the outcome, and whether the gene-environment equivalence assumption is reasonable. Use causal language carefully, clarifying that IV estimates may provide causal effects only under certain assumptions | 11–13 | Biological context is discussed; lifelong cis-eQTL effects are not equated with acute intervention effects. |
|  | c) | Clinical relevance: Discuss whether the results have clinical or public policy relevance, and to what extent they inform effect sizes of possible interventions | 11–13 | No therapeutic readiness is claimed; independent replication and experimental validation are required. |
| 17 | **Generalizability** | Discuss the generalizability of the study results (a) to other populations, (b) across other exposure periods/timings, and (c) across other levels of exposure | 12–13 | Generalizability limits cover ancestry, diabetes context, LD/allele frequency, exposure timing, and eQTL effect scale. |
|  | **OTHER INFORMATION** |  |  |  |
| 18 | **Funding** | Describe sources of funding and the role of funders in the present study and, if applicable, sources of funding for the databases and original study or studies on which the present study is based | 18 | Funding sources and the absence of funder involvement are declared. |
| 19 | **Data and data sharing** | Provide the data used to perform all analyses or report where and how the data can be accessed, and reference these sources in the article. Provide the statistical code needed to reproduce the results in the article, or report whether the code is publicly accessible and if so, where | 14–15 | Data and code availability statements provide the Zenodo DOI, archive names/checksums, environment, scripts, and access limits. |
| 20 | **Conflicts of Interest** | All authors should declare all potential conflicts of interest | 18 | The authors declare no competing interests. |

This checklist is copyrighted by the Equator Network under the Creative Commons Attribution 3.0 Unported (CC BY 3.0) license.

1.	Skrivankova VW, Richmond RC, Woolf BAR, Yarmolinsky J, Davies NM, Swanson SA, et al. Strengthening the Reporting of Observational Studies in Epidemiology using Mendelian Randomization (STROBE-MR) Statement. JAMA. 2021;under review.

2.	Skrivankova VW, Richmond RC, Woolf BAR, Davies NM, Swanson SA, VanderWeele TJ, et al. Strengthening the Reporting of Observational Studies in Epidemiology using Mendelian Randomisation (STROBE-MR): Explanation and Elaboration. BMJ. 2021;375:n2233.
