# Supplementary material for: Kidney-compartment genomic prioritization across diabetic and population kidney traits: an exploratory Mendelian randomization and colocalization study
Source: Front Genet. 2026 Oct 28;17:1921914. doi: 10.3389/fgene.2026.1921914 (PMC13752862; doi:10.3389/fgene.2026.1921914)
Supplement: Supplementary file 4 [file Table14.docx]

# Supplementary Material Manifest

Target journal: Frontiers in Genetics; article type: Original Research.

This revised manifest lists supplementary files prepared for the Frontiers in Genetics specialty-editor revision package. Each file is supplied separately as editable source data, a reviewer-facing reproducibility guide, a standalone MR reporting summary, or a completed official STROBE-MR reporting checklist.

| **File label** | **Title** | **Description** |
| --- | --- | --- |
| Supplementary Data S1 | Functional and translational support | Open Targets, HPA, and INTERVAL-derived tractability and functional support for the final seven-gene panel. |
| Supplementary Data S2 | Disease-state kidney single-cell support | Final-panel gene support from DKD versus control, all disease versus control, CKD versus control, injured proximal tubule trajectory, and fibrotic microenvironment resources. |
| Supplementary Data S3 | Secondary disease-state scan summary | Context sensitivity scan of a second external DKD single-cell supplement for final-panel gene overlap; absence from a source list is not treated as a negative result. |
| Supplementary Data S4 | Progression summary | Resource-level results for CKDGen and KP4CD progression outcomes across GTEx and kidney-compartment resources. |
| Supplementary Data S5 | Progression gene summary | Gene-level summary of recurrent and FDR-significant progression support. |
| Supplementary Data S6 | Gene-level colocalization summary | Maximum PP4 and colocalization class for prioritized genes across tested tissues and outcomes. |
| Supplementary Data S7 | Integrated seven-gene priority panel | Manuscript-facing Table 1 source data and supporting-evidence workbook for disease axis, progression, colocalization, cell-state and functional layers. |
| Supplementary Data S8 | MR safeguards and interpretation limits | Reporting-oriented safeguards for best-instrument MR, sensitivity interpretation, instrument strength, and claim boundaries. |
| Supplementary Data S9 | Independent DKD disease-state expression context | Targeted expression-context assessment of final-panel genes across GSE30528, GSE30529, and GSE142025, including dataset metadata, assay coverage, differential-expression summaries, gene-level context summary, and methods notes; this is not causal validation. |
| Supplementary Data S10 | Colocalization and specificity checks | Prior sensitivity, window-level checks, and supporting evidence for tissue-locus colocalization interpretation. |
| MR reporting summary | MR reporting summary | Standalone MR reporting summary for rapid editorial/statistical review. The same summary is also included in Supplementary Data S8 so that MR safeguards, data-source access information, and reporting notes remain together in one transparency workbook. |

## Major-revision additions

| **File label** | **Title** | **Description** |
| --- | --- | --- |
| Supplementary Data S11 | Final-candidate evidence scope and instruments | Diabetes-specific versus general kidney-trait evidence map; representative SNP, F statistic, exposure/outcome beta and SE, harmonization direction, MR estimate, P value, within-outcome/branch/study-wide FDR, outcome source, selection path, and downgrade rules. |
| Supplementary Data S12 | Study-wide FDR sensitivity | BH-FDR sensitivity across all 597,099 MR screening rows, including universe counts, resource-outcome summaries, and final-panel rows. Correlated and repeated branches are explicitly flagged. |
| Reproducibility Quickstart | Reviewer-facing reproducibility and archive guide | Direct Zenodo DOI, archive filename and checksum, file-manifest and environment locations, minimal verification commands, package file map, and explicit limits on redistribution and full raw-data reruns. |
| **STROBE-MR checklist** | Completed official STROBE-MR checklist | Official EQUATOR/STROBE-MR reporting checklist completed with manuscript page locations, relevant text summaries, transparent not-applicable entries, and explicit identification of analyses that were not performed. |
